# A glycosylated recombinant subunit candidate vaccine consisting of *Ehrlichia ruminantium* major antigenic protein1 induces specific humoral and Th1 type cell responses in sheep

**DOI:** 10.1371/journal.pone.0185495

**Published:** 2017-09-28

**Authors:** Bonto Faburay, Jodi McGill, Frans Jongejan

**Affiliations:** 1 Department of Diagnostic Medicine/Pathobiology, College of Veterinary Medicine, Kansas State University, Manhattan, Kansas, United States of America; 2 Utrecht Centre for Tick-Borne Diseases, FAO Reference Centre for Ticks and Tick-Borne Diseases, Faculty of Veterinary Medicine, Utrecht University, Yalelaan 1, Utrecht, The Netherlands; 3 Department of Veterinary Tropical Diseases, Faculty of Veterinary Science, University of Pretoria, Private Bag X04, Onderstepoort, South Africa; Universidad Nacional de la Plata, ARGENTINA

## Abstract

Heartwater, or cowdriosis, is a tick-borne disease of domestic and wild ruminants that is endemic in the Caribbean and sub-Saharan Africa. The disease is caused by an intracellular pathogen, *Ehrlichia ruminantium* and may be fatal within days of the onset of clinical signs with mortality rates of up to 90% in susceptible hosts. Due to the presence of competent tick vectors in North America, there is substantial risk of introduction of heartwater with potentially devastating consequences to the domestic livestock industry. There is currently no reliable or safe vaccine for use globally. To develop a protective DIVA (differentiate infected from vaccinated animals) subunit vaccine for heartwater, we targeted the *E*. *ruminantium* immunodominant major antigenic protein1 (MAP1) with the hypothesis that MAP1 is a glycosylated protein and glycans contained in the antigenic protein are important epitope determinants. Using a eukaryotic recombinant baculovirus expression system, we expressed and characterized, for the first time, a glycoform profile of MAP1 of two Caribbean *E*. *ruminantium* isolates, Antigua and Gardel. We have shown that the 37–38 kDa protein corresponded to a glycosylated form of the MAP1 protein, whereas the 31–32 kDa molecular weight band represented the non-glycosylated form of the protein frequently reported in scientific literature. Three groups of sheep (*n* = 3–6) were vaccinated with increasing doses of a bivalent (Antigua and Gardel MAP1) rMAP1 vaccine cocktail formulation with montanide ISA25 as an adjuvant. The glycosylated recombinant subunit vaccine induced *E*. *ruminantium-*specific humoral and Th1 type T cell responses, which are critical for controlling intracellular pathogens, including *E*. *ruminantium*, in infected hosts. These results provide an important basis for development of a subunit vaccine as a novel strategy to protect susceptible livestock against heartwater in non-endemic and endemic areas.

## Introduction

Heartwater or cowdriosis is a tick-borne disease caused by an intracellular rickettsial pathogen *Ehrlichia ruminantium* and transmitted by ticks of the genus *Amblyomma*. The disease can cause high mortality of up to 90% in susceptible ruminants [1]. Heartwater is endemic in sub-Saharan Africa where it presents a serious constraint to livestock improvement programs [1]. Through its occurrence in the Caribbean [2,3,4], there is significant threat of introducing heartwater into North America, where the presence of competent vectors, *A*. *cajennense*, *A*. *maculatum* and *A*. *dissimile* have been confirmed [5,6,7].

There is no safe or reliable vaccine available. Due to safety concerns associated with the production and manufacture of live attenuated and inactivated vaccines, subunit vaccines are considered appropriate to control the disease in endemic and non-endemic areas [8]. Antigenic diversity amongst different isolates of *E*. *ruminantium*, resulting in lack of protection between heterologous strains, is the single major obstacle to heartwater vaccine development [8,9,10]. However, the observation that inactivated vaccines stimulate protective immunity [11,12,13] indicates that the development of a successful recombinant vaccine, using selected *E*. *ruminantium* genes, is possible. The Major Antigenic Protein 1 (MAP1), encoded by *map*1 gene, is an immunodominant surface protein of *E*. *ruminantium* and a target for subunit vaccine development. However, sequence polymorphism of the gene among different isolates [14] suggests that any vaccine based on this gene would have to include variants of all important strains [8]. For the American continent, variants of these genes should ideally be obtained from strains in the Caribbean, which, due to geographic proximity, pose the greatest threat of imminent introduction. Previous studies using MAP1 as a subunit vaccine involved DNA vaccine constructs encoding MAP1 of the Crystal Springs isolate from Zimbabwe [15,16]. This vaccine formulation induced partially protective T_H1_ type immune responses in mice; and a subsequent study to improve protection involved boosting with recombinant MAP1 (rMAP1) resulting in survival rate of mice up to 67%. Protection was associated with induction of T_H1_ type immune responses characterized by production of IgG2a and IgG3 anti-MAP1 specific antibodies [15]. However, when delivered as recombinant protein, MAP1 induced a less protective immune response, which was characterized by anti-MAP1 antibodies of predominantly IgG1 isotype [15]. Although, the mechanism by which the *map*1 DNA vaccine elicits protection is not fully elucidated, it clearly involves utilization of the host (eukaryotic) expression machinery to generate MAP1 antigen that induces the host anti-pathogen immune response.

MAP1 has been demonstrated to be a glycoprotein, and the protein is expressed in glycosylated form in eukaryotic (endothelial cells) expression systems [17]. Glycosylation of immunodominant surface proteins has also been reported in other *Ehrlichia* species, whereby weak immunoreactivity has been reported for unglycosylated immunodominant proteins of *E*. *canis* (gp36) and *E*. *chaffeensis* (gp47) [18,19,20]. This suggests that glycans are important epitope determinants. The rMAP1 protein used in prime-boost studies were expressed in a prokaryotic (*E*. *coli*) expression system, which is known to produce nonglycosylated proteins [21]. We postulate that lack of protein glycosylation could account for the suboptimal protective immune response observed in mice vaccinated with the rMAP1 protein. The overall hypothesis is that vaccination with glycosylated forms of rMAP1 proteins produced in a eukaryotic (baculovirus) expression system will elicit a more robust immune response that will confer protection against virulent *E*. *ruminantium* challenge. We have recently reported the successful use of a baculovirus expression system to produce Rift Valley fever virus (RVFV) glycoproteins as constituents of an efficacious vaccine against RVFV infection in a target host [22]. Here, we used a similar baculovirus expression system to produce glycosylated MAP1 proteins from *E*. *ruminantium* and report for the first time the immunogenicity of a glycosylated rMAP1 subunit vaccine in sheep.

## Materials and methods

### Production of recombinant MAP1 protein

#### Cloning and recombinant protein expression

A recombinant baculovirus expression system was used to express glycosylated MAP1 proteins of the *E*. *ruminantium* isolates, Antigua and Gardel. Briefly, the complete *map*1coding sequences of both isolates were codon-optimized including insertion of a Kozak sequence to achieve high level of target protein expression in *Spodoptera frugiperda*, Sf9, insect cells. The sequences were synthesized and cloned into pUC57 vector by a manufacturer (Genewiz). The resulting recombinant plasmid, pUC57-*map*1 was used as template in high fidelity PCR using a proof-reading DNA polymerase, Accuprime DNA polymerase (Life Technologies) and gene-specific primers BYF101F 5′-CACCATGAACTGCAAGAAGATCTTCATCACCTCC-3′ and BYF102R 5′-GAACACGAAACGACCACCGATCTCGATACC-3′. The PCR was performed per manufacturer’s instruction. The PCR amplicon was cloned into pFastBac/CT/TOPO to create donor plasmids, pFastBac-Antigua and pFastBac-Gardel, containing *map*1 coding sequences of Antigua and Gardel, respectively. The recombinant donor plasmids were used to create recombinant bacmid via site-specific transpositioning following transfection into an *E*. *coli* strain, DH10Bac per manufacturer’s instruction (Life Technologies). Recombinant bacmids were purified using HiPure Plasmid kit (Life Technologies) and used to transfect Sf9 cells to rescue respective recombinant baculoviruses. Transfection of recombinant bacmid into Sf9 cells was performed using Cellfectin® reagent per manufacturer’s instruction (Life Technologies). Recombinant baculoviruses, P2 and above, were used to express recombinant *E*. *ruminantium* MAP1 in Sf9 cells.

#### Recombinant protein purification

Recombinant MAP1 proteins were expressed with a C-terminal 6xHis-tag fusion protein that allowed purification by affinity chromatography using Ni-NTA superflow resin (Novagen, Rockland, MA). Purification was performed per manufacturer’s instruction. Briefly, recombinant baculovirus-infected Sf9 cells expressing recombinant *E*. *ruminantium* MAP1 proteins were pelleted by centrifugation at 500 x g for 5 min. The pellet was resuspended in Ni-NTA binding buffer (300 mM NaCl, 50 mM Na_3_PO_4_, pH 8.0, and 10 mM imidazole) containing 1x complete protease inhibitor. Insect Popculture Reagent (Novagen) was then added at 0.05 volumes of original culture volume. The lysate was incubated at room temperature for 15 min and the supernatant further clarified by centrifugation at 1,500 x g for 10 min. The clarified lysate was mixed with previously equilibrated Ni-NTA superflow resin (Novagen-EMD Millipore, Billerica, MA). Binding was performed for 1 hour at 4°C. The suspension was loaded into a column and washed with 10 volumes of wash buffer (300 mM NaCl, 50 mM Na_3_PO_4_, pH 8.0, and 20 mM imidazole). Bound protein was eluted with elution buffer (300 mM NaCl, 50 mM Na_3_PO_4_, pH 8.0, and 250 mM imidazole). The eluate was dialyzed overnight against storage buffer, phosphate-buffered saline (PBS; pH 7.4), and 5% glycerol. Concentration of the purified proteins was determined using the bicinchoninic acid (BCA) assay (ThermoScientific) at an absorbance of 562 nm, using bovine serum albumin (BSA; Sigma-Aldrich) as the protein standard. Aliquots were stored at– 80°C until used.

### Protein analysis

#### Western blot

To assess expression of the target protein, approximately 5 μg of each purified recombinant protein was subjected to electrophoresis in 12% Bis-Tris polyacrylamide gel in 1x MOPS running buffer (Life Technologies). The proteins were transferred onto PVDF membranes per standard protocols. The membrane was blocked in blocking solution containing 0.1% Tween-20 in PBS (pH 7.4) and 3% bovine serum albumin (BSA) for 1 hour at room temperature or overnight at 4°C. After 3 subsequent washes of 5 min each in 0.1% Tween-20 in PBS, the membrane was incubated with anti-His-(C-Terminal)-HRP monoclonal antibody (Life Technologies) diluted 1: 5,000 in blocking solution. Expression of the rMAP1 was further confirmed using mouse anti-*E*. *ruminantium* monoclonal antibody, 4F10B4 (Abcam) [23] at a dilution of 1: 2,000. Following three washing steps, the membrane was probed with a secondary antibody conjugate, goat anti-mouse IgG-HRP (1: 5,000) (Santa Cruz, Biotechnologies). Detection of specific reactivity was performed using ECL enhanced chemiluminescent detection system.

#### Analysis of protein glycosylation

To analyze glycosylation of rMAP1 proteins, approximately 50 μg of purified rMAP1was pelleted at 100,000 x g for 30 min at 4°C. The pellet was resuspended in 2 μl of 10x denaturing buffer (New England Biolabs, Ipswich, MA) and 18 μl distilled water. An untreated rMAP1 protein was included as a negative control. The mixtures were heat-denatured at 100°C for 10 min. Thereafter, the following reagents were added to the reaction: 4 μl of 10x reaction buffer, 4 μl 10% NP40, 4 μl distilled water and 6 μl PNGase F (500,000 U/ml) (New England Biolabs). The mixture was incubated at 37°C for 1 hour and the reaction stopped by addition of SDS loading buffer. The samples were separated by sodium dodecyl sulfate polyacrylamide gel electrophoresis (SDS-PAGE) in NuPAGE 12% Bis-Tris gels (LifeTechnologies) and transferred onto polyvinylidenedifluoride (PVDF) membranes per standard protocols. The membranes were probed with mouse anti-*E*. *ruminantium* monoclonal antibodies as described above. Anti-mouse-HRP conjugated secondary antibody (Santa Cruz Biotechnology) was used for detection. Detection of differentially migrating protein bands (treated and untreated rMAP1) was performed to determine glycosylation or non-glycosylation of the MAP1 proteins. Additional glycosylation analysis of rMAP1 was performed by glycan staining using Pierce in-gel glycan staining protocol (Pierce Thermo Scientific) per manufacturer’s instructions.

### Vaccine preparation

Recombinant *E*. *ruminantium* subunit vaccine was composed of purified glycosylated rMAP1 of Caribbean *E*. *ruminantium* isolates, Gardel and Antigua (a bivalent vaccine). To prepare the vaccine formulations, purified rMAP1 proteins from either isolate were mixed in equal ratios in three different doses: (i) 200 μg (low), (ii) 400 μg (intermediate) and (iii) 600 μg (high). The vaccine was adjuvanted with an montanide ISA25 VG (Seppic, France) per manufacturer’s instruction.

### Animals and vaccination

Twenty sheep (Dorper x Katahdin cross) aged 3–4 months were purchased from a private farm in Kansas and assigned to 4 groups. Group A (*n* = 5), group B (*n* = 6), group C (*n* = 6) and Group D (*n* = 3). Pre-vaccination blood was collected in EDTA from all animals and tested by nested pCS20 PCR to confirm their *E*. *ruminantium*-free status [24]. To assess the appropriate dose response, the vaccine was administered subcutaneously in three different doses. Sheep in group A were vaccinated with 200 μg of rMAP1 subunit vaccine, group B vaccinated with 400 μg, and group C with 600 μg. Group D served as mock-vaccinated control and received adjuvant only. At 21 days post-vaccination, each sheep was boosted with the same vaccine dose with adjuvant administered previously. Animals were bled weekly for serum on day 0 (pre-vaccination), days 7, 14, 21, 28, 35 and 42 post-vaccination. Additionally, at 35 post vaccination, blood was collected in acid citrate dextrose (ACD) tubes for isolation of peripheral blood mononuclear cells (PBMCs) to measure T cell responses as described below. Injection sites and rectal temperatures were examined daily until day 3 post-vaccination for vaccine induced reactions.

### Ethics statement

Research was performed per Institutional Animal Care and Use Committee-approved protocol of Kansas State University (IACUC #3675) in compliance with the Animal Welfare Act and other regulations relating to animals and experiments involving animals. The study was approved by the Institutional Animal Care and Use Committee of Kansas State University.

### Detection of vaccine-induced antibody responses

Induction of MAP1-specific antibody responses was measured in the animals to assess vaccine-induced seroconversion. An indirect ELISA format using recombinant MAP1 of Antigua and Gardel isolates as diagnostic antigens was used in separate assays. Briefly, flat-bottom 96-well microtiter plates (Nunc, MaxiSorp) were coated with 150 ng per well of recombinant MAP1 antigen in 100 μl of antigen coating buffer (Dulbecco’s phosphate buffered saline, DPBS, pH 7.3) and incubated overnight at 4°C. The plates were blocked with PBS pH 7.3, supplemented with 0.1% Tween-20 and 1% non-fat dry milk before adding 100 μl per well of diluted (1:200) serum. Each sample was tested in duplicate and each plate contained duplicate negative and positive control sera obtained from an experimentally infected sheep at Utrecht University in The Netherlands). After washing, plates were incubated with Protein G-HRP (Abcam), diluted 1: 50,000 in blocking solution, at 37°C for 1 hour. Thereafter, 100 μl of substrate buffer containing 0.1 mg/ml 3,3′,5,5′-tetramethyl-benzadine (TMB) (ThermoScientific) and H_2_O_2_ was added and plates were incubated in the dark for 25 min. The reaction was stopped with 2M H_2_SO_4_ and optical densities (OD) were measured at 450 nm using microplate reader Fluostar Omega (BMG Labtech). For each experimental group, the cut-off OD value was determined by addition of 2 standard deviations to the mean OD value of serum obtained from the animals prior to vaccination.

### IgG isotype response

IgG isotype profiling was performed by determining the end titers of IgG1 and IgG2. Mean endpoint titer was determined for animals in group C (vaccinated with the highest vaccine dose 600 μg of rMAP1 subunit vaccine) using day 28 post vaccination sera. Briefly, 5-fold serial dilutions of test serum were initially made and then incubated with rMAP1 antigen (Gardel) coated in 96-well ELISA plates as described above. After three washings, the plate was incubated for 1 hour with mouse anti-bovine IgG1-HRP (Cat. No. MCA2440P; 1:100 dilution) or mouse anti-bovine IgG2 (Cat. No. MCA626; 1:100) (BioRad); both antibodies showed cross-reactivity with sheep. The mouse anti-bovine IgG2 was further probed with Protein G-HRP (1:50,000) for 1 hr. After washing, reactivity was detected by addition of TMB and H_2_O_2_. The reaction was stopped with 2M H_2_SO_4_ and OD values measured at 450 nm as described above.

### Measurement of T-cell responses

Antigen-specific CD4^+^ and CD8^+^ T cell proliferation and cytokine production was measured in peripheral blood on day 35 post vaccination using protocols we have previously published, modified for use in sheep [25,26]. Briefly, peripheral blood mononuclear cells (PBMCs) were isolated by density centrifugation, labeled with Cell Trace Violet proliferation dye (Life Technologies) and placed in culture for 6 days with 1 μg/ml of purified rMAP1. Pokeweed mitogen was included as a positive control and used at 1 μg/ml. On day 6, cells were labeled with the Live/Dead Fixable Aqua Dead Cell Stain Kit (Invitrogen) per manufacturer’s instructions. Samples were then stained with 10 μg/ml mouse anti-ovine CD4 (clone GC1A, isotype IgG2a), and mouse anti-ovine CD8 (clone CACT80C, clone IgG1), both from Washington State University. After washing, samples were labeled with 5 μg/ml of the following secondary antibodies: anti-mouse IgG2a-Alexa Fluor 488 and anti-mouse IgG1-Alexa Fluor 647 (both from Southern Biotech). Cells were then collected on a BD LSR Fortessa X20 Flow Cytometer and analyzed using FlowJo Software (Treestar Inc.). Live cells were gated based upon expression of CD4^+^ or CD8^+^ and proliferation was assessed by dilution of the CellTrace Violet dye. T cell responses were measured by comparing responses to stimulation with rMAP1 from the respective *E*. *ruminantium* strains to mock-stimulated cultures and results are presented as change-over mock. For intracellular cytokine production, PBMC were stimulated overnight with 1 μg/mL *E*. *ruminantium* rMAP1-specific antigen in the presence of 10 μg/mL brefeldin A. After 18–24 hours, cells were surface stained as above. After washing, cells were then fixed with the BD Cytofix/Cytoperm kit per manufacturer’s instructions and stained with 10 μg/mL mouse anti-ovine IFNγ-PE (BioRad Antibodies). Cells were analyzed via flow cytometry as above, gating on live, IFNγ^+^ CD4 and CD8 T cells. The frequency of antigen-specific cells was determined by comparing responses to stimulation with rMAP1 from the respective *E*. *ruminantium* strains to mock-stimulated cultures, and results are presented as change-over mock.

### Statistics

Statistical analysis was performed using Prism V6.0f software (Graphpad Software, Inc.). Differences in antibody responses at various post-vaccination time-points between the vaccine-dose groups in response to either vaccine antigens, Antigua or Gardel, were analyzed using a 2-way repeated measures ANOVA. T cell responses were analyzed using a 1-way ANOVA followed by Tukey’s Multiple Comparisons post-test analysis.

## Results

### Expression of glycosylated recombinant MAP1 proteins

**Fig 1:**
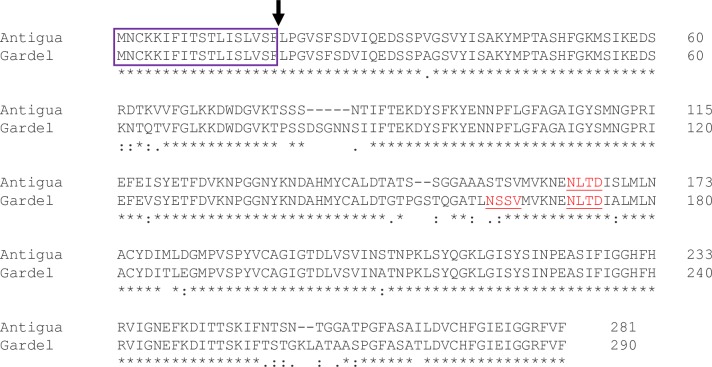
Amino acid sequence alignment of *E*. *ruminantium* MAP1, Antigua (Acc. No. U50830) and Gardel (Acc. no. U50832). Predicted N- and O-linked glycosylation sites are underlined. Rectangular box indicates signal peptide sequence; arrow indicates signal peptidase cleavage site.

**Fig 2:**
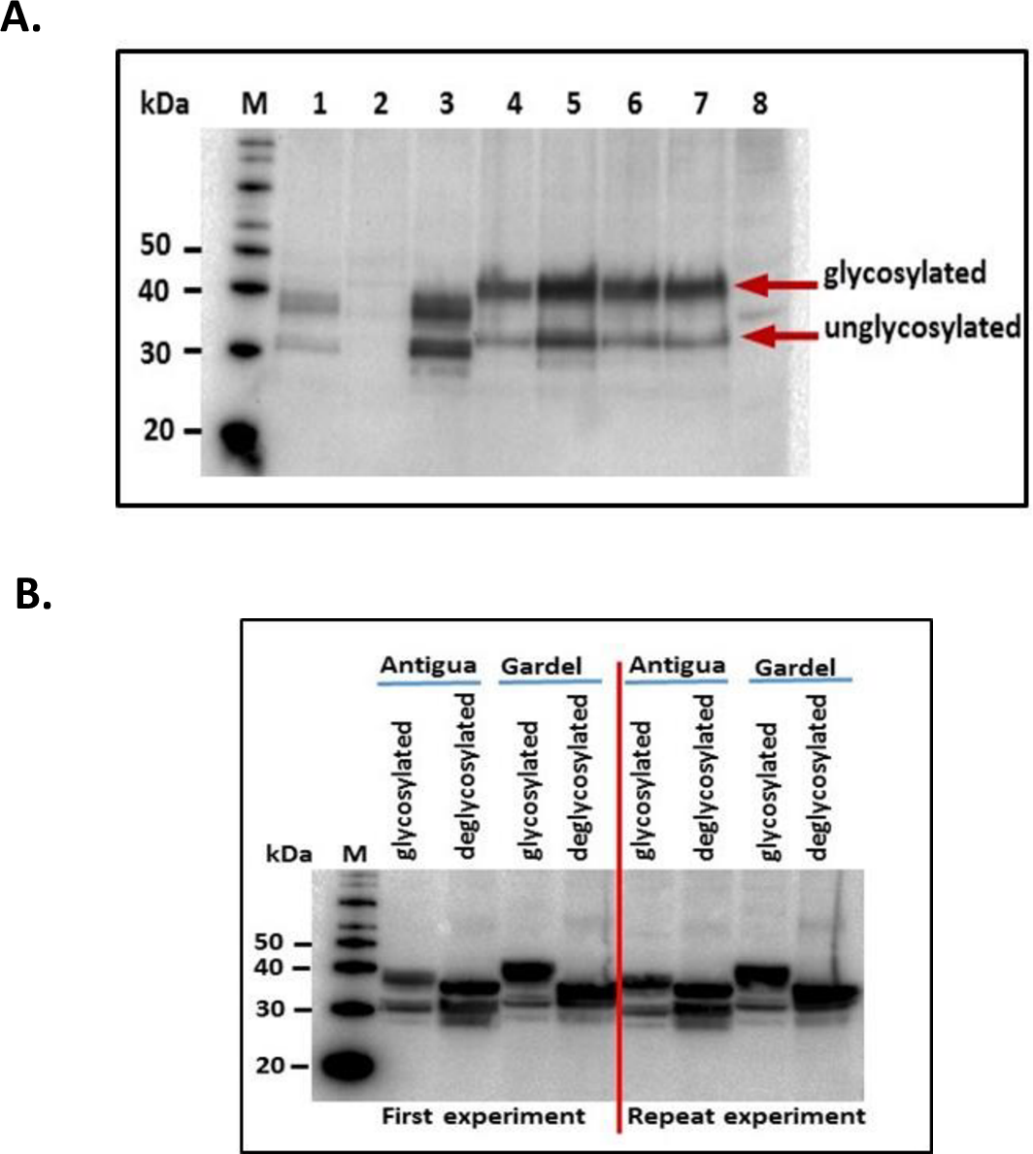
Analysis of recombinant baculovirus expression and glycosylation profile of MAP1 of *E*. *ruminantium* strains, Antigua and Gardel. A. shows detection of expression of rMAP1 by various recombinant baculovirus clones (Antigua: lanes 1,2,3; Gardel: lanes 4,5,6,7; lane 8 –uninfected cell culture control) using mouse anti-His-HRP monoclonal antibody. B. shows glycosylated and deglycosylated forms of the protein following enzymatic treatment of the recombinant protein. Detection was performed using mouse anti-*E*. *ruminantium* monoclonal antibody [23].

*In silico* analysis of MAP1 amino acid sequences of Antigua and Gardel *E*. *ruminantium* isolates revealed the presence of putative sites for N- and O-linked glycosylation (Fig 1). To ensure that rMAP1 proteins were expressed in glycosylated form, cloning of *map1* gene sequences included signal peptide sequences at the N-terminus of the coding sequence (Fig 1), which allowed translocation of the protein into the lumen of endoplasmic reticulum where signal peptidases and glycosylation enzymes are located [27,28,29]. To rescue respective recombinant baculoviruses for expression of rMAP1 proteins, purified recombinant bacmids encoding *map*1 genes of either isolate, Antigua and Gardel, were used to transfect Sf9 cells. The recombinant baculoviruses expressed the MAP1 proteins as demonstrated by detection of 32–33 kDa MAP1 proteins of *E*. *ruminantium*, and a previously undescribed 37–38 kDa protein using anti-His-HRP conjugated and anti-*E*. *ruminantium* monoclonal antibodies (Fig 2A and 2B). To confirm that the recombinant MAP1 proteins of both isolates were expressed in glycosylated form, a deglycosylation assay was performed. Enzymatic de-glycosylation treatment of rMAP1 proteins of both E. *ruminantium* isolates resulted in reduction in the molecular weight of the upper band demonstrated by a shift in electrophoretic migration (Fig 2B). The upper band corresponded to a 37–38 kDa glycosylated form of the immunodominant MAP1 (Fig 2B), whereas the 31–32 kDa molecular weight band represented a non-glycosylated form of the protein (Fig 2B). An in-gel glycan staining confirmed glycosylation of the rMAP1 protein of both *E*. *ruminantium* isolates as indicated by the appearance of specific magenta bands in the stained gel (data not shown).

### Analysis of serological response

**Fig 3:**
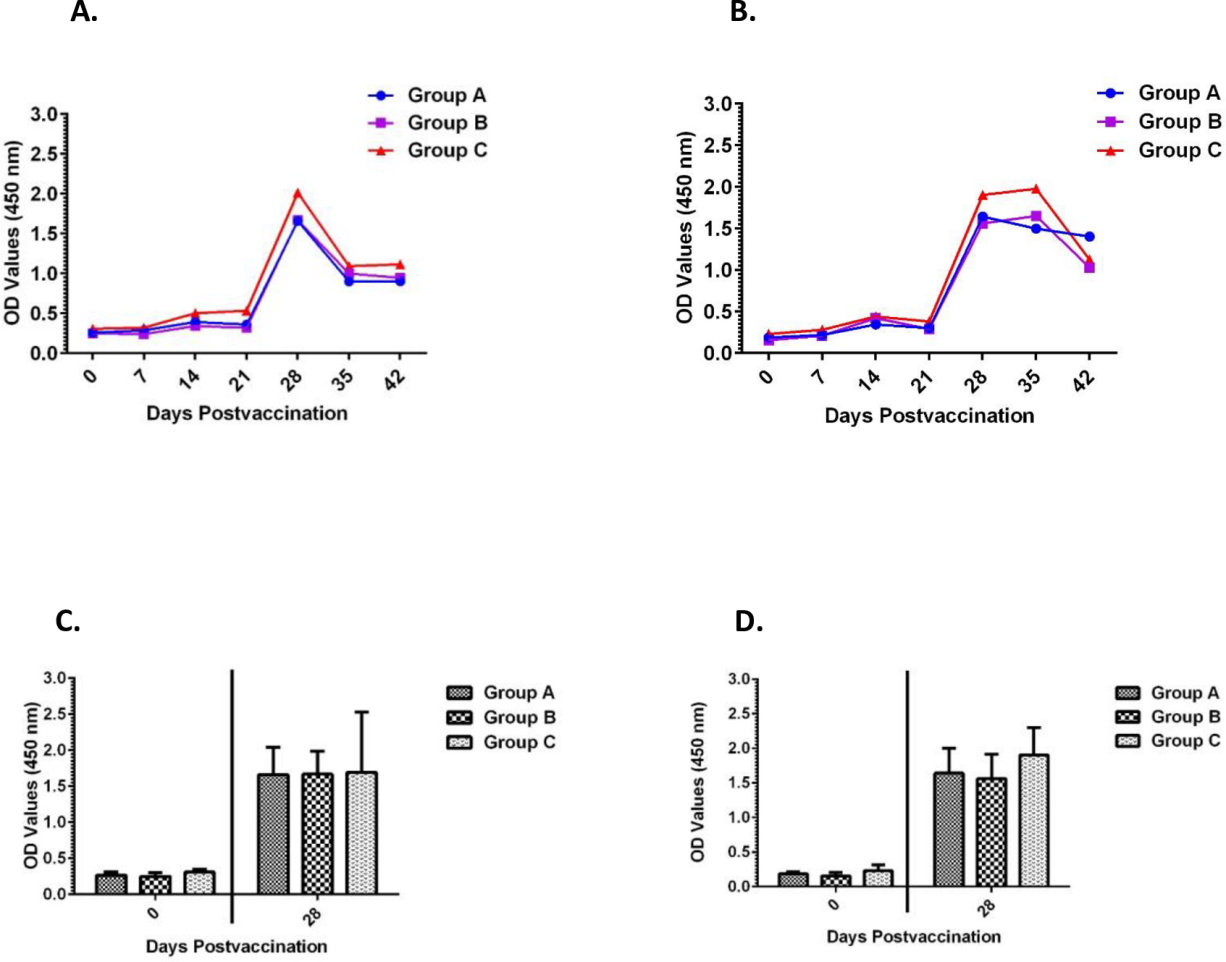
Analysis of MAP1-specific IgG responses in vaccinated sheep by indirect ELISA. A) shows kinetics of antibody responses in sheep vaccinated with Antigua rMAP1; B) shows antibody responses in sheep vaccinated with Gardel rMAP1. MAP1-specific antibody titers persisted through 42 dpv, the study endpoint. C, D) demonstrates the magnitude of rMAP1-specific antibody activity/seroconversion in 28 dpv sera as compared to 0 dpv sera in response to either vaccine antigens, Antigua (left panel) and Gardel (right panel). The cut-off points for Antigua MAP1-specific ELISAs: Group A = 0.237; Group B = 0.254; Group C = 0.356; Gardel MAP1-specific ELISAs: Group A = 0.354; Group B = 0.361; Group C = 0.391.

To assess vaccine induced seroconversion and the kinetics of the antibody response, sera were collected from the vaccinated sheep at various time points post-vaccination (day 0, 7, 14, 21, 28, 35, 42) and were tested MAP1-specific indirect ELISAs. In response to vaccination with the three vaccine dose regimens (Group A = 200 μg, Group B = 400 μg and Group C = 600 μg in equal ratios of rMAP1 proteins of Antigua and Gardel isolates) of the recombinant subunit vaccine, 100% of the animals in all three dose categories seroconverted to both vaccine antigens, Antigua and Gardel rMAP1. Specifically, at day 14 post vaccination all vaccinated animals in the three vaccine dose groups exhibited detectable seroconversion to both vaccine antigens (Fig 3A and 3B). A strong anamnestic response occurred at 28 dpv, which corresponded to peak antibody activity, following administration of a booster at 21 dpv (Fig 3A and 3B). Detectable MAP1-specific antibody titers were maintained in animals in all dose groups until day 42, the study endpoint (Fig 3A and 3B). In response to both vaccine antigens, optical density (OD) values detected at various post-vaccination time-points for the different dose groups were not significantly different (*P* = 0.0606 response to Antigua antigens; *P* = 0.4227, response to Gardel antigen). The magnitude of seroconversion to either vaccine antigen was manifested by marked differences in the absorbance (OD) values in 28 dpv sera (P < 0.05) (Fig 3C and 3D).

**Fig 4:**
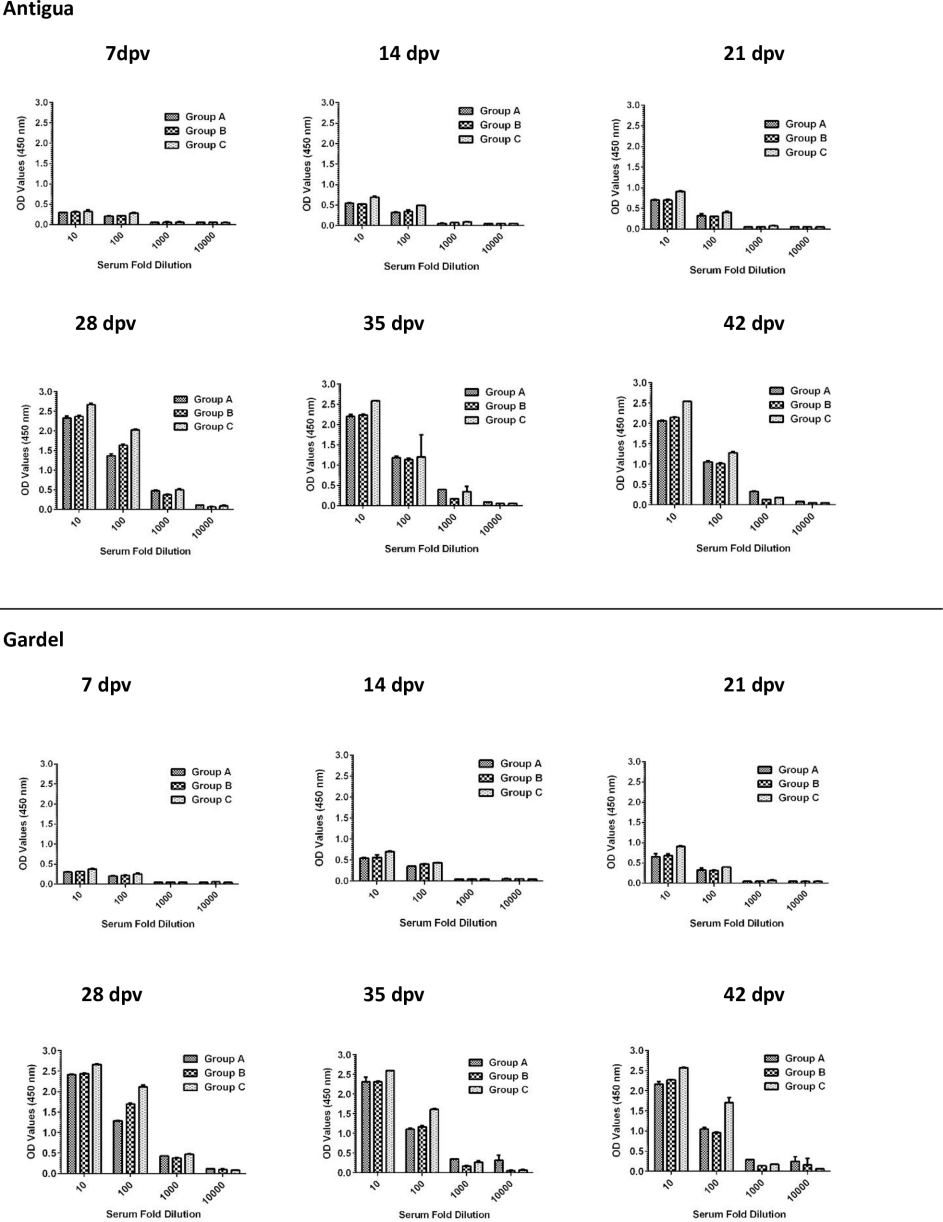
Shows endpoint titration of MAP1-specific antibodies in sera of sheep vaccinated with rMAP1 subunit vaccine. The kinetics of antibody response shows a time-dependent increase in antibody titers for all dose groups, with 28 dpv sera showing the highest endpoint titers in response to both vaccine antigens.

**Fig 5:**
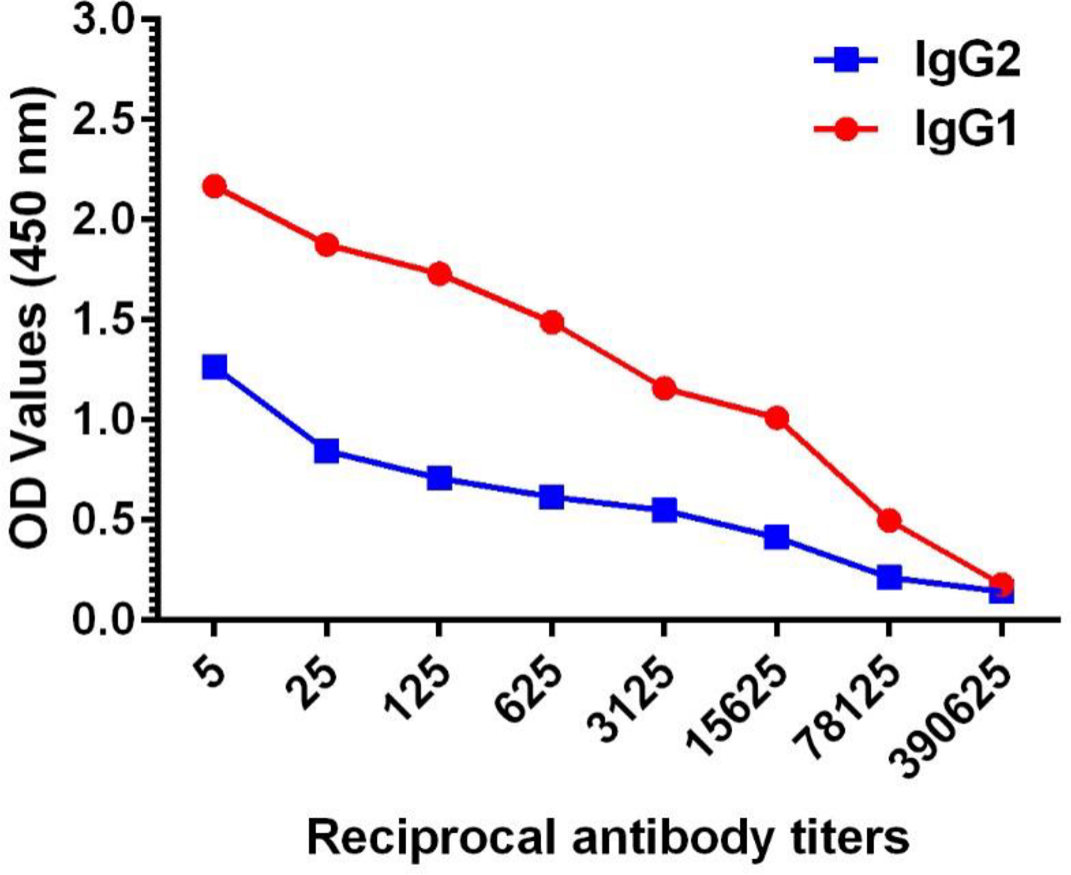
Titers of IgG1 and IgG2 isotypes in sera from sheep vaccinated with rMAP1 subunit vaccine. Mean titers of 28 dpv sera from group D sheep vaccinated with Gardel rMAP1 antigen are shown.

Endpoint titration of MAP1-specific antibody activity in all three vaccine dose groups exhibited a time-dependent increase in antibody titers, with 28 dpv sera showing highest endpoint titers (Fig 4). Overall, antibody reactivity data showed persistence of MAP1-specific antibody titers in all vaccinated animals through the study endpoint. Characterization of immunoglobulin G (IgG) isotype responses in vaccinated sheep showed induction of both IgG1 and IgG2 antibody responses, with the IgG1 isotype showing comparatively higher titers as determined by endpoint titration (Fig 5).

### Analysis of CD4^+^ and CD8^+^ T cell responses

**Fig 6:**
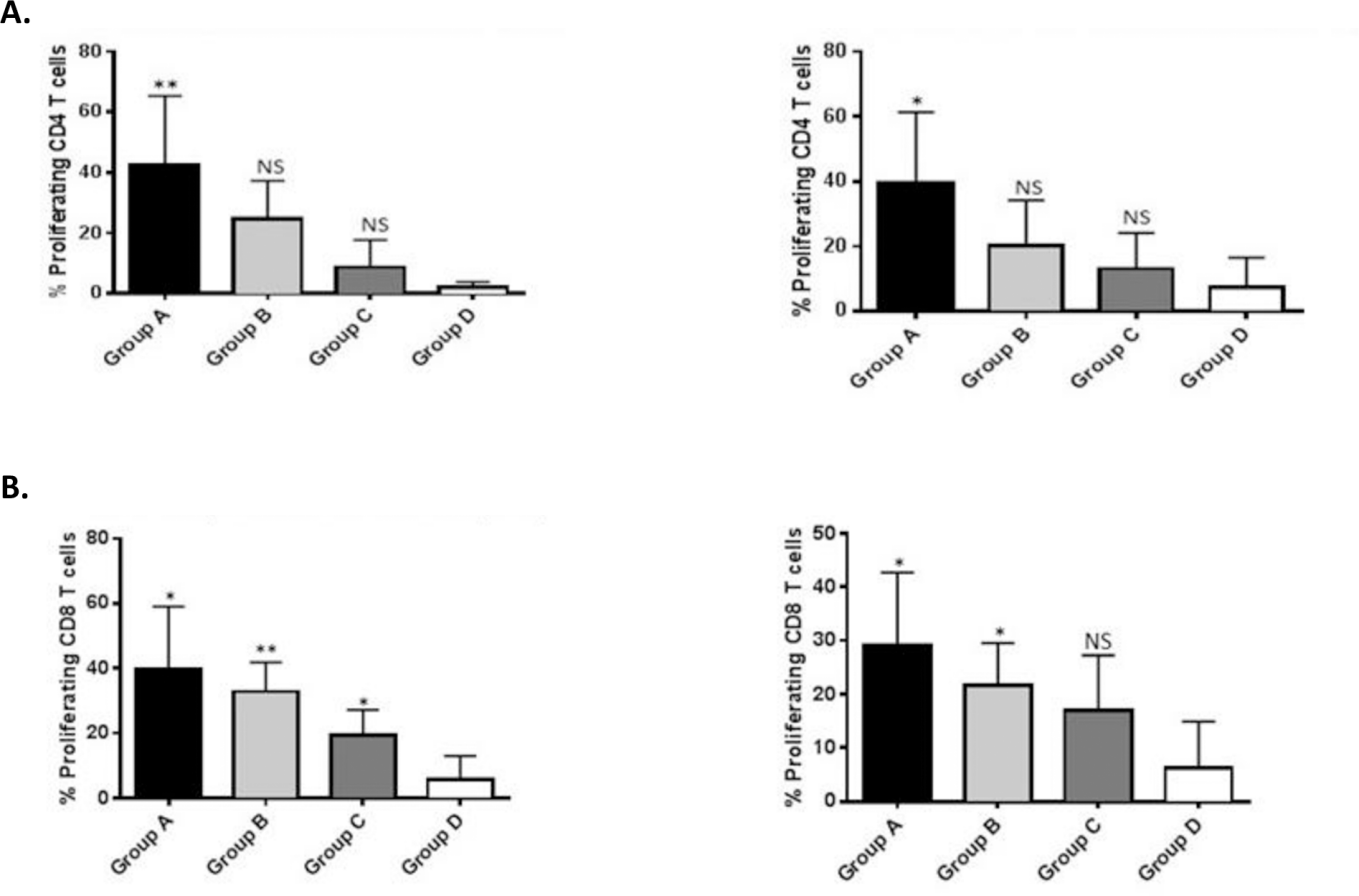
Measurement of T cell proliferation in response to rMAP1 subunit vaccination in sheep. A) Upper panel shows CD4^+^ T cell proliferation following antigenic stimulation with Antigua (upper left panel) or Gardel (upper right panel) rMAP1 proteins. B) Bottom panel shows CD8^+^ T cell proliferation in response to rMAP1 antigen stimulation with Antigua (bottom left panel) or Gardel (bottom right panel) rMAP1 proteins. Asterisks denote statistically significant; NS = not significant.

PBMC isolated from the vaccinated animals demonstrated significant antigen-specific CD4^+^ and CD8^+^ T cell proliferation in recall response to either recombinant antigen (Antigua or Gardel rMAP1) (Fig 6A and 6B). Animals vaccinated with 200 μg (group A) of rMAP1 demonstrated the highest frequency of proliferating CD4^+^ T cells compared to mock vaccinated animals (group D) when stimulated with rMAP1 of either *E*. *ruminantium* isolates, Antigua or Gardel (*P* < 0.05) (Fig 6A). The frequency of proliferating CD4^+^ T cells detected in animals in group B (vaccinated with 400 μg of subunit vaccine) and group C (vaccinated with 600 μg subunit vaccine) was not statistically different from the mock-vaccinated control group D (P > 0.05) (Fig 6A). Furthermore, vaccinated animals in groups A, B and C exhibited a higher frequency of proliferating CD8^+^ T cells following antigenic stimulation with Antigua rMAP1 than mock-vaccinated control group D (P < 0.05) (Fig 6B). Vaccinated animals in groups A and B also exhibited higher percentage proliferating CD8^+^ T cells than the mock control group D in response to stimulation with Gardel rMAP1 antigen (P < 0.05) (Fig 6B); however, the frequency of proliferating CD8^+^ T cells from animals in group C were not statistically different from the mock-vaccinated control group D (P > 0.05) (Fig 6B). Comparison of the frequency of proliferating CD4^+^ or CD8^+^ T cells between the various vaccine-dose groups (A, B, C) showed no statistically significant differences (P > 0.05).

**Fig 7:**
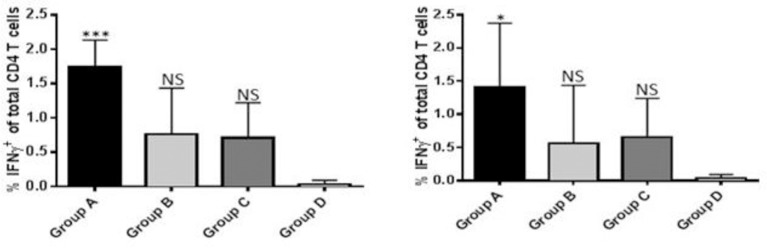
Analysis of IFN-γ production by CD4^+^ T cells isolated from sheep vaccinated with rMAP1 subunit vaccine. Antigenic stimulation was performed using Antigua (left panel) and Gardel (right panel) rMAP1 antigens. Asterisks denote statistically significant; NS = not significant.

To assess antigen-specific IFNγ production, CD4^+^ T cells were subjected to intracellular cytokine staining following antigenic stimulation with Antigua or Gardel rMAP1. Group A animals responded with higher percent of IFNγ-positive CD4^+^ T cells than the mock-vaccinated control group D (P < 0.05) (Fig 7). The frequency of IFNγ-positive CD4^+^ T cells detected in groups B and C animals was higher but statistically not different from the control group D animals (P > 0.05) (Fig 7). Comparison of the percent IFNγ-positive CD4^+^ T cells between the various vaccine-dose groups (A, B, C) revealed no statistically significant differences (P > 0.05). Similarly, a comparison of the frequency of IFNγ-positive CD8^+^ T cells revealed no statistically significant differences between the various vaccine-dose groups (data not shown).

## Discussion

Current vaccines developed and evaluated for protecting ruminant livestock against heartwater are based on inactivated [12,13,24,30,31,32,33] and live-attenuated vaccines [24,34,35]. These vaccines suffer from low immunogenicity (for killed vaccines), lack of cross-protection and/or safety concerns, especially for use in non-endemic areas. Beside a few sheep studies [36,37,38], most subunit vaccine studies were performed in mouse models [15,16,38,39]. This represents the first study wherein the immunogenicity of glycosylated forms of the major antigenic protein 1 (MAP1) of *E*. *ruminantium* is assessed in a ruminant model. The *E*. *ruminantium* MAP1 is an immunodominant membrane protein and considered a good target for vaccine development. Like most membrane proteins with potential sites for glycosylation [40], *in silico* analysis of *E*. *ruminantium map*1 gene revealed putative *N*-linked and *O*-linked glycosylation sites (Fig 1). *O*-linked sites have been reported to serve as sites for *O*-linked mannosylation, which has been shown, in eukaryotes, to enhance immunogenicity of membrane proteins [40]. In this study, we confirmed expression of *E*. *ruminantium* rMAP1 (Antigua and Gardel isolates) protein in glycosylated form utilizing a eukaryotic expression system and demonstrated a distinct glycosylation profile of the protein (Fig 2). Vaccination of sheep with a subunit vaccine formulation induced both antibody and Th1 T cell responses, which are critical to controlling intracellular pathogens, including *E*. *ruminantium*, in infected hosts; suggesting that a glycosylated rMAP1 subunit vaccine could be potentially efficacious against virulent heartwater challenge.

Specifically, the recombinant MAP1 subunit vaccine induced a strong Th1 T cell response characterized by increased proliferation of CD4^+^ and CD8^+^ T cells, including production of IFN-γ, and induction of IgG2 isotype antibodies. The significance of this cellular response in terms of protective immunity in the ruminant host is unknown, as we were unable to perform efficacy testing in this study. However, in a mouse model, a polarized Th1 response characterized by induction of IgG2a and IgG3 antibodies, following a prime-boost vaccination regimen (DNA prime and *E*. *coli*-expressed rMAP1 boost), was associated with increased protection against virulent heartwater challenge [15]. We postulate that increased protection is attributed to *in vivo* (eukaryotic) expression of glycosylated MAP1 from the DNA vector construct, since immunization with unglycosylated *E*. *coli*-expressed MAP1 proteins resulted in lower protective efficacy characterized by a Th2 response of predominantly IgG1 isotype [15]. Indeed, the potential role of glycans in antigenicity has been reported for glycoproteins of *E*. *ruminantium* (MAP1), and antigenically related pathogens, *E*. *canis* (gp36) and *E*. *chaffeensis* (gp47), for which glycosylated forms of the proteins exhibited stronger immunoreactivity in comparison to unglycosylated forms [17,18,19]. The enhanced T cell response observed in our study could be attributed to the presence of *O*-linked glycans, by facilitating recognition by mannose receptors on dendritic cells, resulting in efficient uptake, processing, and presentation of antigen to T cells [40]. Taken together, these results suggest that glycan residues in *E*. *ruminantium* MAP1 proteins are important antigenic determinants. Further studies are required to support this hypothesis including the potential ability to confer broad-spectrum protection amongst antigenically diverse strains of the pathogen.

Although immunity to *E*. *ruminantium* infection (heartwater), like other intracellular parasites, is mainly cell-mediated [41,42], the role of the humoral immune response has not been fully elucidated. In this study, the recombinant MAP1 subunit vaccine also induced strong anamnestic MAP1-specific antibody response in sheep; and even though antibodies produced in response to *E*. *ruminantium* infection have been reported to not correlate with protective immunity against heartwater [42], we speculate that their induction is an important vaccine attribute and could play a role as opsonizing agents and contribute to enhancing cell-mediated immunity.

With the aim of assessing the dosage of the subunit vaccine that induces enhanced immune response in sheep, we tested three doses (low, intermediate and high) of the vaccine. There was no significant difference between the different dose groups in inducing antibody responses, as assessed by the level of IgG antibody responses (*P* > 0.05) (Fig 3). In contrast, the dose of the antigen/vaccine appeared to influence the level of cellular responses; showing vaccination with a low dose inducing higher frequency of proliferating CD4+ and CD8+ T cells, including higher levels of secreted cytokine, IFN-γ, compared to vaccination with intermediate and high doses. The lack of positive correlation is unknown; however, studies in mice have shown that the dose of antigen may be a critical factor in determining what type of immune response will be elicited; with susceptible mice immunized with very low doses of *Leishmania majo*r demonstrating higher levels of protection than did mice immunized with higher doses of the organism [43]. It has been postulated that very small doses of antigen could imprint the immune response into a Th1-like cell-mediated mode or select T cells with high functional avidity, whereas higher immunizing doses inhibit this response [44, 45]. It is unknown if the comparatively low T cell response observed in the intermediate and high-dose vaccinated groups could be attributed to a similar phenomenon.

## Conclusions

This study represents an important first step towards developing an efficacious heartwater subunit vaccine and defining the immunological correlates potentially associated with protection in ruminants, the target host species. Although we were not yet able to perform efficacy studies (due to lack of permit and virulent *E*. *ruminantium* isolate), the induction of strong Th1 type cellular and antibody responses in vaccinated animals is encouraging. Th1 helper T cells express IFN-γ, which has been shown to be essential for early protection against a range of intracellular pathogens by contributing to macrophage activation and other cell-mediated responses [46,47,48]. Given these results and the emerging threat of heartwater to livestock agriculture and food security, it is necessary to perform further studies to assess the efficacy of the subunit vaccine in experimental sheep.
